# A randomised trial of adaptive pacing therapy, cognitive behaviour therapy, graded exercise, and specialist medical care for chronic fatigue syndrome (PACE): statistical analysis plan

**DOI:** 10.1186/1745-6215-14-386

**Published:** 2013-11-13

**Authors:** Rebecca Walwyn, Laura Potts, Paul McCrone, Anthony L Johnson, Julia C DeCesare, Hannah Baber, Kimberley Goldsmith, Michael Sharpe, Trudie Chalder, Peter D White

**Affiliations:** 1 MH&N Clinical Trials Unit, Institute of Psychiatry, King’s College London, DeCrespigny Park, London SE5 8AF, UK; 2 Centre for the Economics of Mental Health, Institute of Psychiatry, King’s College London, DeCrespigny Park, London SE5 8AF, UK; 3 MRC Biostatistics Unit, Institute of Public Health, University Forvie Site, Robinson Way, Cambridge CB2 0SR, UK; 4 MRC Clinical Trials Unit, Aviation House, 125 Kingsway, London WC2B 6NH, UK; 5 University Department of Psychiatry, Warneford Hospital, Oxford OX3 7JX, UK; 6 Department of Psychological Medicine and Psychiatry, King’s College London, Weston Education Centre, Cutcombe Road, London SE5 9RJ, UK; 7 Centre for Psychiatry, Wolfson Institute of Preventive Medicine, Barts and the London School of Medicine and Dentistry, Queen Mary University of London, London EC1A 7BE, UK; 8 Clinical Trials Research Unit, Leeds Institute of Clinical Trials Research, University of Leeds, Leeds LS2 9JT, UK

**Keywords:** Statistical analysis plan, chronic fatigue syndrome, myalgic encephalomyelitis, randomised controlled trial, PACE trial

## Abstract

**Background:**

The publication of protocols by medical journals is increasingly becoming an accepted means for promoting good quality research and maximising transparency. Recently, Finfer and Bellomo have suggested the publication of statistical analysis plans (SAPs).The aim of this paper is to make public and to report in detail the planned analyses that were approved by the Trial Steering Committee in May 2010 for the principal papers of the PACE (Pacing, graded Activity, and Cognitive behaviour therapy: a randomised Evaluation) trial, a treatment trial for chronic fatigue syndrome. It illustrates planned analyses of a complex intervention trial that allows for the impact of clustering by care providers, where multiple care-providers are present for each patient in some but not all arms of the trial.

**Results:**

The trial design, objectives and data collection are reported. Considerations relating to blinding, samples, adherence to the protocol, stratification, centre and other clustering effects, missing data, multiplicity and compliance are described. Descriptive, interim and final analyses of the primary and secondary outcomes are then outlined.

**Conclusions:**

This SAP maximises transparency, providing a record of all planned analyses, and it may be a resource for those who are developing SAPs, acting as an illustrative example for teaching and methodological research. It is not the sum of the statistical analysis sections of the principal papers, being completed well before individual papers were drafted.

**Trial registration:**

ISRCTN54285094 assigned 22 May 2003; First participant was randomised on 18 March 2005.

## Update

### Background

#### ***Publication of statistical analysis plans***

The review and publication of study protocols by medical journals are increasingly becoming an accepted means for promoting good quality research and maximising transparency. Since 1997 *The Lancet* has actively invited investigators to submit their protocols to the journal for peer review, offering a provisional commitment to publish the principal results where their criteria are satisfied [1-4]. Since 2001, following a call from Chalmers and Altman [5], *BioMed Central* has been inviting trialists and other researchers to publish their full protocols online [6]. *The British Medical Journal*, while not offering peer review or publication as yet, has required authors to submit trial protocols with their manuscripts since January 2005, making them available to editors and reviewers as additional documentation [7]. More recently, calls have been made for the publication of other key trial documentation. Chan, for instance, has argued the case for public access to regulatory agency submissions [8]. In an editorial for *Critical Care and Resuscitation*, Finfer and Bellomo suggested the publication of statistical analysis plans [9]. The plans for the NICE-SUGAR (Normoglycaemia in Intensive Care Evaluation and Survival Using Glucose Algorithm Regulation) and RENAL (Randomised Evaluation of Normal versus Augmented Level of Replacement Therapy) studies [10,11] were published in the same issue.

A statistical analysis plan (SAP) is defined within the International Conference on Harmonisation’s guidance on the statistical principles for clinical trials (ICH E9) as ‘a document that contains a more technical and detailed elaboration of the principal features of the analysis described in the protocol, and includes detailed procedures for executing the statistical analysis of the primary and secondary variables and other data’ [12]. According to ICH E9, the statistical analysis plan should be pre-specified, completed after the protocol has been finalised but reviewed and possibly updated as a result of a blind review of the data carried out after the completion of data collection. It is suggested that details of the primary analysis should be clearly distinguished from those of supporting analyses and that the methods for handling missing data, outliers and multiplicity be described [12]. While the statistical analysis plan is clearly an important document, at present it is rarely made available to people outside of the study.

There are many reasons why study-specific statistical analysis plans should be published in full, with electronic journals offering the greatest potential for this to be commonplace. Due to space constraints, the paper providing the principal results often contains only a very limited description of the analyses that were planned or carried out. If the study protocol is published, further information is likely to be available. However, this is often insufficient to enable full replication of the analyses. The statistical analysis plan complements both the protocol and the principal paper by providing a systematic and comprehensive description of the planned analyses, taking into consideration any relevant methodological or clinical developments that may have arisen since the study’s inception. Its publication enables any changes to the original plan to be laid out, increasing the scientific rigour and transparency with which the principal analyses are currently reported.

Maximum transparency regarding what decisions were made *a priori* could be achieved by publishing the statistical analysis plan, which has been approved by the Trial Steering Committee (TSC), before the results of a study are known. The final analyses reported may differ from those planned, allowing for *post*-*hoc* analysis where it is indicated (as Finfer and Bellomo [9] have noted), reporting alternative methods if statistical models do not converge, and omitting planned analyses that are superseded, redundant, or no longer of interest. Assessment of the validity of the analyses, reporting and consequent interpretation would also be made easier by the increased visibility of selective or misreporting. This may, in turn, encourage more balanced, accurate and complete reporting of results and ultimately help to raise the standard of trial analyses. Peer review has particular advantages, as it encourages dialogue, the quality of which is likely to be improved by the level of detail given. Knowledge of this added scrutiny should, in turn, act to promote the quality of the submitted plan. This process would be especially valuable if the research is anticipated to generate debate or if it might have a large impact on clinical practice.

The benefits of publication go beyond those specific to the study. Making statistical analysis plans accessible will help future statisticians and other researchers design and analyse better studies. This is because each study throws up different issues, often more complex than the standard textbook ones. Publishing details of the ways in which different groups choose to address these helps to generate discussion and could also promote greater communication and collaboration between methodologists, applied statisticians and researchers.

#### ***The PACE trial***

The rationale for the trial is outlined in the protocol [13] and main clinical paper [14]. To be brief, chronic fatigue syndrome is characterised by chronic disabling fatigue in the absence of an alternative diagnosis, present in 0.2 to 2.6% of the population. The National Institute for Health and Clinical Excellence (NICE, UK) recommends two treatments: cognitive behaviour therapy (CBT) and graded exercise therapy (GET), but patient organisations recommend a third treatment: adaptive pacing therapy (APT). A definitive randomised trial was therefore needed to compare all three treatments with specialist medical care (SSMC) and to compare the established treatments (CBT, GET) against the new treatment (APT).

The objective of this paper was to make public and report in detail the planned analyses for the principal papers of the PACE (Pacing, graded Activity, and Cognitive behaviour therapy; a randomised Evaluation) trial, using the template statistical analysis plan developed by the Mental Health and Neuroscience (MH&N) Clinical Trials Unit based at the Institute of Psychiatry. These planned analyses were written with a view to publication and are reproduced almost as they were approved by the Trial Steering Committee (Version 1.2 dated 2 May 2010) prior to database lock. The changes from the original document were editorial clarifications suggested by reviewers and editors for which we are most grateful; these changes in no way alter the strategy for analysis. The SAP supplements the published protocol [13], the main clinical [14] and health economics [15] papers and the authors’ reply [16] to a selection of correspondence published by the Lancet [17-24]. They also provide an illustration of the planned analyses of a complex intervention trial taking into account the impact of clustering by care providers, where multiple care providers are present for each patient in some but not all arms of the trial. Details of the statistical aspects of multiple therapist-per-patient designs are published elsewhere [25].

### Statistical analysis plan

#### ***Introduction***

##### 

**Purpose and scope of statistical analysis strategy** This document details the presentation and analysis strategy for the principal paper(s) reporting results from the PACE Trial. It is intended that the results reported in these papers will follow the strategy set out here; subsequent papers of a more exploratory nature will not be bound by this strategy but will be expected to follow the broad principles laid down for the principal papers. The principles are not intended to curtail exploratory analysis or to prohibit sensible statistical and reporting practices, but they are intended to establish the strategy that will be followed, as closely as possible, when analysing and reporting the trial. Reference was made to the published trial protocol [13], ICH Guidance on Statistical Principles for Clinical Trials (E9) [12], CPMP points to consider on multiplicity [26], and CONSORT guidelines for the reporting of harms [27] and for non-pharmacological treatment trials [28].

##### 

**Analysis strategy group** The Statistical Analysis Strategy was developed by the PACE Analysis Strategy Group whose members were:

– Michael Sharpe (Chair, Principal Investigator)

– Rebecca Walwyn, Laura Potts, Tony Johnson and Kim Goldsmith (Statisticians)

– Paul McCrone (Health Economist)

– Peter White and Trudie Chalder (Principal Investigators)

– Julia DeCesare and Hannah Baber (Trial Managers)

##### ***Convention***

Throughout this Statistical Analysis Strategy the four individual randomised interventions are referred to as APT (adaptive pacing therapy *plus* standardised specialist medical care), CBT (cognitive behaviour therapy *plus* standardised specialist medical care), GET (graded exercise therapy *plus* standardised specialist medical care), and SSMC (standardised specialist medical care *alone*).

Unless stated otherwise ‘intervention’ refers to the four randomised interventions (group), and ‘therapy’ refers to APT, CBT, or GET. ‘Treatment’ is used more generally and embraces all forms including drugs.

The anchoring date for visits and assessments is randomisation; thus 24 weeks refers to 24 weeks from randomisation.

#### ***Trial design and objectives***

##### 

**Study objectives** The PACE trial aims to answer the questions set out below under primary objectives, secondary objectives, and health economics objectives.

**Primary objectives**:

1. Is APT more effective than SSMC in reducing (i) fatigue or (ii) disability up to 52 weeks from randomisation?

2. Is CBT more effective than SSMC in reducing (i) fatigue or (ii) disability up to 52 weeks from randomisation?

3. Is GET more effective than SSMC in reducing (i) fatigue or (ii) disability up to 52 weeks from randomisation?

4. Is CBT more effective than APT in reducing (i) fatigue or (ii) disability up to 52 weeks from randomisation?

5. Is GET more effective than APT in reducing (i) fatigue or (ii) disability up to 52 weeks from randomisation?

**Secondary objectives**:

1. Is the pattern of results relating to the primary objectives replicated with the outcome as the participants’ self-rated clinical global impression change rating?

2. Do different interventions have differential effects on the two primary outcomes (that is, fatigue versus disability)?

3. Are the differences across interventions in the primary outcomes associated with similar differences in secondary outcomes?

Health economics objectives

The primary health economics objectives are as indicated below:

1. To compare care costs (including the costs falling to health service agencies, other agencies and also those borne by patients and their carers) and lost-employment costs up to 52 weeks for (i) CBT versus APT; (ii) GET versus APT; (iii) SSMC versus APT; (iv) CBT versus SSMC; (v) GET versus SSMC; and (vi) CBT versus GET.

2. To assess the relative cost-effectiveness and cost-utility of APT, CBT, GET, and SSMC (with costs based on health, social care, and informal care) up to 52 weeks.

The secondary health economics objectives are as indicated below:

1. To compare care costs (including the costs falling to health service agencies, other agencies and also those borne by patients and their carers) and lost-employment costs between randomisation and 24 weeks for (i) CBT versus APT; (ii) GET versus APT; (iii) SSMC versus APT; (iv) CBT versus SSMC; (v) GET versus SSMC; and (vi) CBT versus GET.

2. To assess the relative cost-effectiveness and cost-utility of APT, CBT, GET, and SSMC (with costs based on health, social care, and informal care) up to 24 weeks.

3. To describe the annual healthcare and societal costs at baseline and their association with clinical and demographic characteristics.

4. To describe and compare patterns of service utilisation up to 24 weeks and up to 52 weeks across the four interventions.

5. To identify patient characteristics which predict service costs for each intervention.

6. To identify patient characteristics which predict cost-effectiveness/cost-utility up to 24 weeks, and up to 52 weeks for each intervention.

Health economic hypotheses

The primary hypotheses are:

1. Health and other service costs do not differ between APT, CBT, and GET up to 24 weeks, and up to 52 weeks, but are all higher than the costs for SSMC.

2. Total (health and societal) costs up to 24 weeks, and up to 52 weeks, are highest for SSMC, followed by APT, and with no substantial difference between CBT and GET.

3. APT has better cost-effectiveness and cost-utility than SSMC up to 24 weeks, and up to 52 weeks.

4. Both CBT and GET have better cost-effectiveness and cost-utility than SSMC and APT up to 24 weeks, and up to 52 weeks, but their cost-effectiveness does not differ substantially.

The secondary hypotheses are:

1. Higher healthcare costs are associated with being female, being older and having comorbid conditions, particularly mood disorders and having other symptom-based diagnoses.

2. Higher total societal costs are associated with being male, being younger, having more severe physical disability, pervasive passivity (measured by actigraphy), certain illness beliefs, and having comorbid conditions, particularly mood disorders and having other symptom-based diagnoses.

##### 

**Outcome measures** The primary outcome measures will be:

1. Fatigue Chalder Fatigue Questionnaire (CFQ) (Likert scoring) [29].

2. Physical Disability SF-36 physical function (SF-36PF sub-scale, Version 2) [30].

Secondary outcome measures include safety outcomes, efficacy outcomes and health economics outcomes.

Safety outcomes are:

1. Serious deterioration (primary) defined as one or more of the following up to 52 weeks:

a. SF-36 physical function score diminishing by 20 or more points between baseline and any two consecutive assessment interviews.

a. Participant-rated CGI change score of “much worse” or “very much worse” at two consecutive assessment interviews.

a. Withdrawal from therapy (APT, CBT, or GET) later than 8 weeks due to participant’s reported worsening of their condition.

a. A serious adverse reaction.

2. Serious adverse events (includes serious adverse reactions and suspected unexpected serious adverse reactions).

3. Serious adverse reactions (includes suspected unexpected serious adverse reactions).

4. Non-serious adverse events (includes non-serious adverse reactions); numbers, proportions, and examples.

5. Withdrawals from the interventions.

The four components of ‘serious deterioration’ will be reported in addition to the composite outcome.

Efficacy outcomes are:

1. Participant rated Clinical Global Impression (CGI) [31] change category.

2. Anxiety measured by HADS-A subscale of the Hospital Anxiety and Depression Scale [32].

3. Depression measured by HADS-D subscale of the Hospital Anxiety and Depression Scale [32].

4. Six-Minute Walking Test [33].

5. Work and Social Adjustment measured by WSAS [34].

6. Participant Satisfaction (7-point item from very satisfied to very dissatisfied).

7. Centers for Diseases Control (CDC) Symptoms - Number of symptoms [35].

8. Jenkins sleep score [36].

A selection of the above efficacy outcomes will be reported in the primary paper as required to aid interpretation of the primary outcomes; other secondary outcomes will be reported in subsequent papers. The selection will, in part, be determined by space constraints.

Health economics outcomes are:

1. CSRI (service, societal, NHS and insurance/benefits costs) [37].

2. EuroQol [38].

##### Trial design

**Figure 1:**
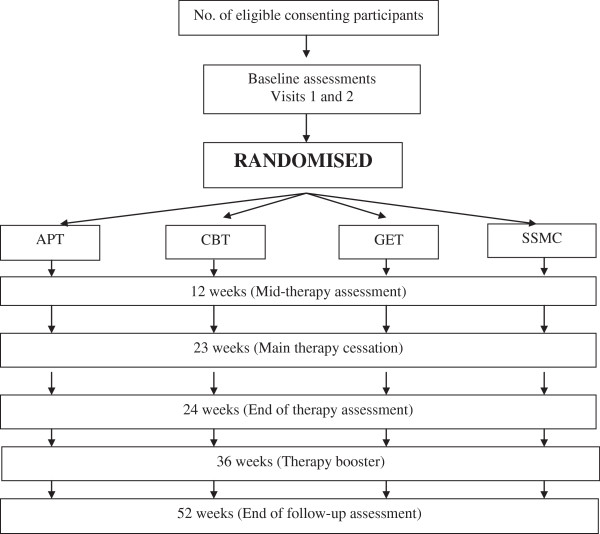
Flow diagram.

Figure 1 gives an overview of the trial design.

##### **Basic design (including sample size)**

Date of First Randomisation: 18 March 2005

Date of Last Randomisation: 28 November 2008

Target for Randomisation: 600

Number Randomised: 641

**Eligibility criteria**:

1. Written informed consent from the participant

2. Clinical diagnosis of CFS based on Oxford research diagnostic criteria

3. Therapy needs make participation appropriate

4. Aged 18 years or over

5. Adequate level of English comprehension

6. Chalder Fatigue bimodal score of 6 or more

7. SF-36 physical function subscale score of 65 or less

8. No psychiatric exclusions listed in the Oxford research diagnostic criteria

9. Able to attend for therapy and research assessments

10. No contraindications to any of the trial interventions

11. No previous trial therapy at a PACE centre

**Randomised interventions (all manuals are available from http://www.pacetrial.org):**

1. Adaptive pacing therapy + standardised specialist medical care (APT).

APT is based on the illness model of CFS/ME as a currently undetermined organic disease, with the assumption that APT can improve quality of life while not affecting the core disease other than providing the best conditions for natural recovery. APT is essentially an energy management approach, which involves assessment of the link between activity and subsequent symptoms and disability, using a daily diary, with advice to plan and pace activity to avoid exacerbations.

2. Cognitive behavioural therapy + standardised specialist medical care (CBT).

CBT is based on the illness model of fear avoidance, used in the three previous trials of CBT [39-41]. There are three essential elements: (a) assessment of illness beliefs and coping strategies; (b) structuring of daily rest, sleep, and activity, with a graduated return to normal activity; and (c) collaborative challenging of unhelpful beliefs about symptoms and activity.

3. Graded exercise therapy + standardised specialist medical care (GET).

GET is based on the illness model of deconditioning and exercise intolerance, used in the previous trials [42,43]. Therapy involves an assessment of physical capacity, negotiation of an individually designed home exercise programme with set target heart rates and times, and participant feedback with mutual planning of the next fortnight’s exercise programme.

4. Standardised specialist medical care alone (SSMC).

SSMC is given to all participants and includes visits to the clinic doctor with general, but not specific advice, regarding activity and rest management, such as advice to avoid the extremes of exercise and rest, as well as symptomatic pharmacotherapy. SSMC is standardised in the SSMC Doctor’s Manual. SSMC participants, like all other participants, will already have received the patient clinic leaflet (PCL). There will be no additional therapist involvement, and, in particular, there will be no diary monitoring with consequent advice.

**Participating centres**:

Table 1 gives the details of the participating centres, including their IDs.

**Table 1:** Details of participating centres

**ID**	**Clinical service**	**Centre leaders**
1	Chronic Fatigue Clinic, St Bartholomew’s Hospital, London	Professor PD White
2	Chronic Fatigue Syndrome Service, Western General and Astley Ainsley Hospitals, NHS Lothian, Scotland	Dr D Wilks, Professor MC Sharpe
3	Chronic Fatigue Research Unit, King’s College Hospital, London	Professor T Chalder, Professor S Wessely
4	Chronic Fatigue Clinic St Bartholomew’s Hospital, London	Dr M Murphy
5	Oxfordshire Mental Healthcare NHS Trust and Oxford Radcliffe Hospitals Trust, Oxford	Dr B Angus, Professor T Peto, Dr E Feldman
6	Fatigue Service Royal Free Hampstead NHS Trust, London	Dr G Murphy
7	Pain Management Centre Frenchay Hospital, Bristol	Dr H O’Dowd

Centres 1 and 4 were combined on 01 June 2006 and are regarded as a single centre for both randomisation and analysis.

##### **Sample size calculation taken from the protocol (v5.2)**

The following is quoted from the PACE trial protocol (v5.2) (see also [13]) and describes sample size estimation based on percentages responding to the trial interventions. The primary outcomes were changed subsequently to measures on continuous scales.

At one year we assume that 60% will respond with CBT, 50% with GET, 25% with APT, and 10% with SSMC. The existing evidence suggests that at one-year follow-up, 50 to 63% of participants with CFS/ME had a positive outcome, by intention to treat, in the three RCTs of rehabilitative CBT [39-41] with 69% improved after an educational rehabilitation that closely resembled CBT [44]. This compares with 18 to 63% improved in the two RCTs of GET [42,43] and 47% improvement in a clinical audit of GET [45]. For usual medical care 6 to 17% improved by one year in two RCTs [40,41]. There are no previous RCTs of APT to guide us, but we estimate that APT will be at least as effective as the control therapy of relaxation and flexibility used in previous RCTs, with 26 to 27% improved on primary outcomes [39,43].

Our planned intention to treat analyses will compare APT against SSMC alone, and both CBT and GET against APT. Assuming α = 5% and a power of 90%, we require a minimum of 135 participants in the SSMC alone and APT groups, 80 participants in the GET group and 40 in the CBT group [46]. However these last two numbers are insufficient to study predictors, process, or cost-effectiveness. We will have low statistical power to detect the difference between CBT and GET, though our estimates will be useful in planning future trials. As an example, to detect a difference in responder rates of 50 and 60%, with 90% power, would require 520 participants per group; numbers beyond a realistic two-arm trial. Therefore, we will study equal numbers of 135 participants in each of the four arms, which gives us greater than 90% power to study differences in efficacy between APT and both CBT and GET. We will adjust our numbers for dropouts, at the same time as designing the trial and its management to minimise dropouts. Dropout rates were 12 and 33% in the two studies of GET [42,43] and 3, 10, and 40% in the three studies of rehabilitative CBT [39-41]. On the basis of our own previous trials we estimate a dropout rate of 10%. We therefore require approximately 150 participants in each intervention group, or 600 participants in all. Calculation of the sample size required to detect economic differences between intervention groups requires data on cost per change in outcome, which are not currently available.

##### 

**Stratification at randomisation** Allocation of interventions to participants was by minimisation with a random component [47] and four stratification factors:

Centre (6 strata): 1 and 4, 2, 3, 5, 6, 7

CDC Criteria (2 strata): Met or unmet

London Criteria (2 strata): Met or unmet

Current Depressive Disorder (2 strata): Present or absent

Participants found to be incorrectly stratified will be kept in their original strata for the primary analysis in accordance with the principle of intention-to-treat (ITT) [48]. The extent of incorrect stratification will be reported.

#### ***Data collection***

##### 

**Screening measures** A clinic patient log book was kept of all new CFS/ME referrals to trial centres to facilitate monitoring of recruitment to the trial. Screening information will not be used in the analysis except for:

1. Reasons for patients not taking part in the trial (see Participant Flow).

2. Chalder Fatigue Questionnaire and SF-36 Physical Functioning subscale scores where these are not available at baseline (see Method for Handling Dropouts and Missing Data).

##### 

**Baseline and outcome measures** The information collected at baseline and follow-up is presented in Table 2.

**Table 2:** Timing of research assessments

	**Baseline**^**a**^	**Week 12**	**Week 24**	**Week 52**	**Discontinuation of therapy**	**Discontinuation of follow**-**up**
**Demographic and clinical data**						
Eligibility	✓					
Centre	✓					
Date of birth	✓					
Gender	✓					
Ethnicity	✓					
Marital status/dependents	✓					
Living arrangements	✓					
Usual place of residence	✓					
Educational level	✓					
Group membership	✓			✓		
Height and weight	✓					
Employment status	✓					
Benefits/pensions status	✓					
Start of current illness	✓					
Start of disabling episode	✓					
Comorbid medical conditions	✓	✓	✓	✓	✓	
Medications and therapies	✓	✓	✓	✓	✓	
Oxford criteria	✓			✓	✓	
CDC criteria/CDC symptoms	✓			✓	✓	
London criteria	✓			✓	✓	
Fibromyalgia	✓			✓	✓	
Past medical history	✓					
Preferred intervention group	✓					
Adverse events		✓	✓	✓	✓	
**Therapist data**^**c**^						
Primary qualification	✓					
Years of experience	✓					
Years of relevant experience	✓					
Employment grade	✓					
**Doctor data**^**c**^						
Discipline	✓					
Employment grade	✓					
**Questionnaires**						
Chalder fatigue	✓	✓	✓	✓	✓	✓
SF-36 physical functioning	✓	✓	✓	✓	✓	✓
Participant CGI		✓	✓	✓	✓	✓
Doctor CGI				✓	✓	✓
Therapist CGI			✓		✓	✓
Walking test	✓		✓	✓	✓	
Actigraphy	✓					
HADS	✓	✓	✓	✓	✓	
Self-efficacy	✓	✓	✓	✓	✓	
WSAS	✓	✓	✓	✓	✓	
SIQ^b^	✓	✓	✓	✓		
PHQ-15	✓	✓	✓	✓	✓	
Exercise and activity	✓	✓	✓	✓	✓	
Jenkins Sleep Scale	✓	✓	✓	✓	✓	
Step test	✓	✓	✓	✓	✓	
Borg Scale	✓	✓	✓	✓	✓	
Participant satisfaction			✓	✓		
SSMC/therapy adherence			✓	✓	✓	✓
EQ-5D	✓		✓	✓	✓	
CSRI	✓		✓	✓	✓	

^a^Baseline was conducted over two research visits prior to randomisation; ^b^The SIQ is now known as the Cognitive and Behavioural Questionnaire; ^c^The therapist and doctor data will be kept separate from the trial database and summarised by the Trial Manager (see Baseline Comparability of Randomised Groups); Note: CDC, Centers for Disease Control; CGI, clinical global impression; CSRI, client service receipt inventory; EQ-5D, Euroqol 5 dimensions; HADS, hospital anxiety and depression scale; PHQ15, physical health questionnaire 15; SF-36, short-form 36; SIQ, symptoms interpretation questionnaire; SSMC, standardised specialist medical care; WSAS, work and social adjustment scale.

In addition, details were recorded for i) training sessions, ii) therapist competency, iii) quality control checks of therapy sessions, and iv) homework compliance assessments that were made after every therapy session and that will be summarized as part of the general description of intended intervention policies.

Primary and secondary outcome variables will be derived from the follow-up data at each relevant time-point as follows.

Primary outcomes:

i) Fatigue total score (Likert scoring, higher scores indicate more fatigue).

ii) Physical disability total score (sum of 10 items multiplied by 5, lower scores indicate more disability).

Secondary outcomes are presented in Table 3.

**Table 3:** Derivation of secondary outcomes

	**Secondary outcome**	**Derivation**
iii)	Serious deterioration	Absent/present - derived using the DMEC algorithm
iv)	Serious deterioration: Component 1	Absent/present - SF-36 physical function score diminishing by 20 or more points between baseline and any two consecutive assessment interviews
v)	Serious deterioration: Component 2	Absent/present - Participant-rated CGI change score of ‘much worse’ or ‘very much worse’ at two consecutive assessment interviews
vi)	Serious deterioration: Component 3	Absent/present - Withdrawal from therapy more than 8 weeks after randomisation due to participant’s reported worsening of their condition
vii)	Serious deterioration: Component 4	Absent/present - A serious adverse reaction
viii)	Serious adverse events	Total number up to 52 weeks
ix)	Serious adverse reactions	Total number up to 52 weeks
x)	Adverse events	Total number and the proportion of participants having one or more up to 52 weeks
xi)	Withdrawals from intervention	No/yes; person responsible; reason; days from randomisation
xii)	Participant-rated CGI	Positive change; no change; negative change
xiii)	Anxiety (HADS-A)	Total (sum) of the anxiety items of the HADS (higher scores indicate more anxiety)
xiv)	Depression (HADS-D)	Total (sum) of the depression items of the HADS (higher scores indicate more depression)
xv)	Six minute walking test	Total number of meters walked - derived from the number of 10 meter lengths plus any partial distance
xvi)	Work and social adjustment	Total (sum) of all items (higher scores indicate less adjustment)
xvii)	Participant satisfaction	Very satisfied; moderately satisfied; slightly satisfied; neither; slightly dissatisfied; moderately dissatisfied; very dissatisfied
xviii)	CDC Symptoms (#)	Total (sum) of CDC symptoms 1 to 8
xix)	Jenkins Sleep Score	
xx)	CSRI - service costs	Total (sum) costs - derived by assigning costs (£) to each relevant item in the CSRI
xxi)	CSRI - societal costs	Total (sum) costs - derived by assigning costs (£) to each relevant item in the CSRI
xxii)	CSRI - NHS costs	Total (sum) costs - derived by assigning costs (£) to each relevant item in the CSRI
xxiii)	CSRI - insurance/benefit costs	Total (sum) costs - derived by assigning costs (£) to each relevant item in the CSRI
xxiv)	EuroQol	Item scores for Q1 to 5 will be weighted by utility values and summed to produce a total (this can range from −0.59 to 1, with 1 indicating full health)

Note: CDC, Centers for Disease Control; CGI, clinical global impression; CSRI, client service receipt inventory; DMEC, data monitoring and ethics committee; HADS, hospital anxiety and depression scale.

##### 

**Trial periods (recruitment and follow-up)** Recruitment was initially intended to be ongoing for 36 months, with three centres recruiting during the first 12 months, six centres recruiting during the subsequent 24 months, and three centres recruiting at twice the annual rate during the last 12 months. Due to a funding extension, a seventh centre (Bristol) was added and recruitment was ongoing for 45 months.

SSMC is ongoing over 52 weeks; therapy in APT, CBT and GET is ongoing for the first 23 weeks with one booster session between 36 and 52 weeks. Participants are followed up at 12, 24, and 52 weeks.

##### 

**Research visit window definitions** Screening data are collected prior to baseline visit 1; baseline data are collected prior to randomisation. Baseline visit 2 is at least one week after baseline visit 1. Baseline CFQ and SF-36PF should be collected within one month prior to randomisation. Follow-up data should be collected within one week of the expected date where possible. Week 52 follow-up data can be collected at any time after week 52 with no specified upper time limit other than the end of 52 week data collection for the trial (31 January 2010).

When research visits fall outside of the guidance window, they will be analysed according to the most appropriate time point. Specifically, planned visits taking place up to 18 weeks will be used for the 12-week data, while the closest planned visit will be used for the 24- and 52-week data. If a planned visit data is missing, previous unscheduled visit data can be used instead.

Visit windows will be summarised to indicate whether their distribution is similar across interventions; the use of unscheduled visits will also be summarised.

Where variation in visit times is large, or the average visit time differs across interventions, time will be fitted as a continuous instead of a categorical variable. This decision will be made by a consensus judgement of the authors.

#### ***General considerations***

##### 

**Blinding of the statistical analysis** This document has been developed without reference to the PACE trial database. No analyses of outcomes relating to this strategy have been, or will be, conducted prior to final written approval of the analysis strategy by the TSC. Reports have been prepared with data presented descriptively by intervention (coded to maintain blinding) for the closed sessions of the Data Monitoring Committee. Consequently, both DMC and TSC were blind to intervention group, as were the trial statisticians. Data cleaning will be performed as blind to intervention allocation as possible. Decisions made during analysis concerning data or additional analyses will be documented.

##### 

**Trial samples** Numbers (and percentages) of participants satisfying the following definitions will be reported overall and by intervention.

##### 

**Intention-to-treat sample** The intention-to-treat (ITT) sample is defined as all participants who were randomised into the trial included in the intervention to which they were randomised, regardless of the presence or absence of follow-up data. Participants will be included in the stratum in which they were randomised.

##### 

**Available-case sample** The available case sample is defined as all participants who were randomised into the trial, who have any outcome data available for analysis, included in the stratum and intervention to which they were randomised. This sample will be a subset of the ITT sample, excluding randomised participants who have no outcome data.

##### 

**Per-protocol sample** The per-protocol sample is defined as all participants who were randomised into the trial, who met trial eligibility criteria, and who followed their randomised intervention policy at the centre in which they were randomised; they will be included in the intervention to which they were randomised and with their correct stratum. This sample will be a subset of the ITT sample, excluding randomised participants who (i) are confirmed not to have met trial eligibility criteria at randomisation, and (ii) departed from their randomised intervention policy at any point up to 52 weeks.

##### 

**As-treated sample** The as-treated sample is defined for the health economic analyses as all participants who were randomised into the trial and received one of the trial interventions. This sample will be a subset of the ITT sample, excluding participants who have not received any of the four interventions. Participants will be assigned to their received therapy rather than to their randomised intervention if these disagree.

##### 

**Safety sample** The safety sample is the ITT sample for this trial.

##### **Other samples**

1. The sample screened for eligibility is defined as all consecutive new outpatients referred to PACE recruiting centres with a possible or definite clinical diagnosis of CFS/ME between 12 October 2004 and 14 November 2008.

2. The sample assessed for eligibility is defined as all patients consenting to formal eligibility assessment by the research workers.

3. The therapist sample includes all the therapists who were assessed for their competency in delivering trial therapies.

4. The doctor sample includes all the doctors signed up to deliver trial interventions.

5. The research worker sample includes all research assistants or research nurses collecting PACE trial data.

#### ***Adherence to the protocol***

##### 

**Blinding of randomised interventions** The members of the Data Monitoring Committee, Trial Steering Committee and the trial statisticians were blinded to intervention allocation. Participants and all other research and therapy staff were not blinded, since it was impossible to do so. The steps taken to minimise and measure bias were:

1. Primary outcomes were self-rated by the participant.

2. Outcome assessments were coordinated by research workers not directly involved in the interventions participants received.

3. Equipoise was actively encouraged throughout the planning and course of the trial.

4. Baseline staff expectations regarding the outcome of the trial were recorded.

5. Participant intervention preferences and expectations regarding the outcome of their intervention were recorded.

##### **Departure from intended therapy (APT, CBT, GET)**

Departures from intended therapy refer to discrepancies between the intended therapy (as described in the therapy manuals) and the manner in which the therapies were actually delivered within the trial. To assess the extent of fidelity to the manuals as well as the distinguishability of the therapies, a random sample of audio recordings of therapy session number 10 will be independently and blindly assessed at the end of the trial. This will be done by competent therapists who do not have specific allegiance to any of the three forms of therapy. The sample will be of sufficient size to ensure that at least one tape from each therapist will be assessed. Each tape will be evaluated by two raters using a treatment integrity schedule specifically designed for the purpose. The scheme will be piloted using three tapes from each therapy, nine in total. Inter-rater reliability will be assessed and the ratings reported using descriptive statistics.

##### 

**Departures from randomised intervention policy** The overall definition of departures from the randomised intervention policy is given in terms of session attendance as:

a. Fewer than three sessions of SSMC (participants allocated SSMC only)

b. Fewer than ten sessions of APT, CBT or GET (participants allocated these therapies)

The number of sessions includes both face-to-face sessions and those conducted over the telephone. Within this definition, formal withdrawal from intervention after three sessions of SSMC or ten sessions of APT, CBT, or GET have been completed will not be regarded as a departure from the randomised intervention policy. However any participant withdrawing from his or her randomised intervention, or initiating another trial therapy prior to the above cut-offs would be regarded as a departure from the randomised intervention (it will be noted when this was by mutual consent). This includes participants randomised to SSMC who, in fact, receive APT, CBT or GET as a trial therapy. The overall compliance variable will therefore be binary separating those who followed their randomised intervention policy from those who did not.

The average (and range) of the numbers of therapy and SSMC sessions attended will be reported by intervention.

##### 

**Withdrawals from intervention** The decision to withdraw a participant from an intervention is made by the clinician or the participant (active withdrawals).

The number of active withdrawals (broken down by initiator (participant, clinical staff, both)) will be reported by intervention and centre, and by interval from randomisation. The most common reasons for withdrawal will be summarised.

##### **Withdrawals from the trial and losses to follow-up**

The decision to withdraw a participant from follow-up within the trial is made only when the participant withdraws their consent to research follow-up. All reasonable attempts are made to continue to follow up all participants, including those that withdraw from intervention.

For the purposes of analysis, losses to follow-up are those missing all primary outcome scale data at all follow-up assessments, those missing all primary outcome scale data at weeks 24 and 52, or those missing all primary outcome scale data at week 52.

The numbers of withdrawals and losses to follow-up will be reported (see Comparisons of Losses to Follow-Up).

#### ***Statistical considerations***

##### 

**Stratification in the analysis** The primary analysis of therapy effect will be adjusted by the factors used for stratification at randomisation (that is, centre, CDC criteria, London criteria and current depressive disorder) [12,49] and by the baseline assessment of the outcome variable.

##### 

**Method for handling centre effects** The PACE trial was designed with variation in participant outcomes between centres rather than between doctors or therapists in mind. For the primary analysis to be consistent with the trial design, the primary method for handling contextual variation in the analysis of therapy effects will be to include centre as a fixed covariate. The centre that randomises the largest number of participants will be the reference category. The centre assigned to each participant will be based on the participant’s centre at randomisation. Consideration will also be given to including centre as a random effect [50].

##### 

**Method for handling other clustering effects** Outcomes at weeks 12, 24 and 52 are nested within participants. The primary method for handling clustering associated with repeated measurements will be to fit a cluster-specific random effects model [51-53] including the participant as a random intercept, and investigating the addition of a random slope over time. Where therapy effects cannot be interpreted as population-averaged effects because outcomes are binary, a population-average (GEE) model will also be fitted.

**Figure 2:**
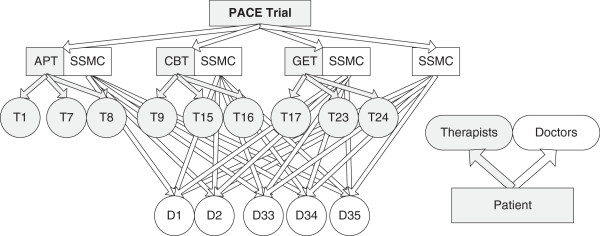
Data structure envisaged in the design.

Due to (i) the nesting of participants within therapists and doctors; (ii) the partial nesting of therapists within APT, CBT, and GET as there was no therapist involvement in SSMC; and (iii) the crossing of doctors with interventions, variation in participant outcomes between therapists, and in intervention effects between SSMC doctors, are recognised to be potential sources of clustering in this trial [54]. The data structure envisaged in the design (Figure 2) differs from that observed in practice due to a number of planned deviations resulting from unavoidable therapist absences (section 8.6 of Protocol v5.2). To summarise:

a. Local centre cover delivered by a PACE therapist of the same discipline working in a nearby centre will mean that some therapists will be crossed with centres.

b. Distant therapy delivered by a PACE therapist of the same discipline means that participants will not always be seen by a single therapist.

c. Cross-cover therapy delivered by a PACE therapist of a different discipline means that participants will not always be seen by a single therapist and some therapists may be crossed with the therapies.

d. Recruitment of a replacement therapist means that more than one therapist per centre may deliver each therapy.

It is also possible that participants may be seen by more than one SSMC doctor over the course of the trial.

These deviations are anticipated to affect less than 10% of the trial participants. We will initially assume independence of outcomes within therapists/doctors in the primary analysis. Two further analyses are planned, using two-level heteroscedastic models assuming a fully nested design [54], with clusters based on i) the main care provider and ii) the pair comprising the main therapist and the main doctor to assess the robustness of the model to the assumption of independence.

If no clustering is found in (i) supporting the conclusions of the primary analysis, then (ii) will not be performed. The ‘main care provider’ is defined as the therapist or doctor providing the largest number of trial therapy sessions for each participant. As such, the main care provider is likely to be a therapist for APT, CBT, and GET and a doctor for SSMC. To be explicit, if the doctor provides more sessions than the therapist in APT, CBT or GET then the doctor is the main care provider (see Departures from Randomised Intervention Policy). If there is a tie in number of sessions delivered by two care providers the main care provider will be the one who delivered the earlier sessions.

In summary, three analyses are planned: 1) without accounting for therapist effect/clustering, 2) accounting for main care provider, and 3) accounting for both the main therapist and main doctor for each participant. The third will not be done if the second shows no clustering effects.

An analysis accounting for the effect of clustering on secondary outcomes will be considered.

Any differences in the point estimates, confidence intervals (CIs) or conclusions will be reported. Any problems encountered in fitting these models will be reported and the scope of the analyses will be restricted; the weights used within the multiple membership model [55] will be determined by the proportion of participants treated by each therapist/doctor.

Additional models to explore or take account of complex clustering effects may also be fitted; if so, the motivation for these will be reported together with their results.

##### 

**Method for handling dropouts and missing data** Data are missing completely at random (MCAR) when they represent a simple random sample of the complete sample and the missing data mechanism is independent of all observed and unobserved variables. The assumption that data are missing at random (MAR) is reasonable when missing data represent an identifiable stratified sample of the complete sample and the missing data mechanism is dependent only on other known and observed variables. Data are missing not at random (MNAR) where missing data represent an unidentifiable stratified sample of the complete sample and the missing data mechanism depends on measured and unmeasured variables. The model describing the missing data mechanism will take any clustering effects into consideration. The planned strategy for handling missing data at the item [56] and scale [57] levels will depend on whether the amount of item-missing data observed is minimal. Within practical constraints it will be assumed that data are missing at random (MAR) conditional on the variables included in the substantive model.

##### 

**Missing item data** To ensure the same strategy is followed across all scales reported in the principal paper(s) any guidance given by authors of validated questionnaires will be superseded by the strategy outlined here. Where item-missing data are considered minimal (defined here as no more than 10% of participants with any missing item data across visits where collected or where no more than 20% of the items within a scale are missing within participants), prorating (that is, mean imputation across items within a scale, or subscale where scales are formed of subscales, for each visit and participant) [56] will be used. The focus will instead be on handling scale-missing data. Any bias or underestimation of variance of scores associated with prorating is anticipated to be negligible where item-missing data are minimal [56]. We will report the amount of missing item data by the percentage of participants who have more than 10% item missing data for each scale reported.

The amount of item-missing data is expected to be minimal. However, if this is not so for any outcome scale then multiple imputation [58,59] at the item-level will be the primary method used. Items will be imputed 100 times [60] separately for each scale (with the exception of the CFQ and SF-36PF, which will be imputed simultaneously). All of the other items for that scale across all time points (including baseline), scores (overall and any subscales) across all time points (including baseline), the four stratification factors at randomisation, randomised intervention, main therapist, and main SSMC doctor will be included in the imputation model.

##### 

**Missing scale data** Missing baseline scale data are not an issue for the primary analysis of efficacy; no missing data are expected for the stratification factors. Where the CFQ or SF-36PF is missing at baseline they will be replaced by the relevant scale at screening. There is specific guidance for missing baseline scale data, and this will be followed [61]. That is, we will use mean imputation of baseline variables assuming baseline and outcome are correlated less than 0.6.

Where the amount of item-missing data is considered minimal, missing outcome scale-data will be handled within the primary analysis by maximum likelihood [57,62] under a similar model for the missing data mechanism assumed for missing item data (see section above). We will report the amount of missing scale data by the percentage of participants who have more than 10% missing item data for each scale reported.

##### ***Loss to follow-up***

Some participants will withdraw from follow-up during the trial, and for these it may be more appropriate to assume data are missing not at random (MNAR). Where more than 10% of randomised participants are lost to follow-up, the impact of this will be investigated in a sensitivity analysis using the weighting approach described by Carpenter, Kenward and White [63] if multiple imputation is the primary method, or comparing selection model and pattern-mixture model therapy effect estimates [64] where maximum likelihood is the primary method.

##### ***Method for handling multiple comparisons and multiplicity***

The overall probability of falsely claiming a statistically significant result increases when multiple significance tests (or equally CIs) are interpreted simultaneously. Multiplicity considerations arise in this trial from the presence of (i) multiple outcomes, (ii) multiple intervention comparisons, and (iii) multiple analyses.

The strategy for adjusting, presenting and interpreting the results is set out below.

Multiplicity adjustments will be made as follows:

1. The following five comparisons will be made using two-sided hypothesis tests (alpha = 0.05) at 52 weeks: APT versus SSMC, CBT versus SSMC, GET versus SSMC, CBT versus APT, GET versus APT.

For the co-primary outcomes, fatigue and disability, and for the secondary outcome, the participant-rated CGI, *P*-values will be presented unadjusted for multiplicity.

2. In addition Bonferroni adjustment (0.05/5) will be applied separately to each of the three outcomes to control the outcome-wise type I error rate at 5%.

3. No adjustment will be made for any sensitivity analysis as their purpose is to increase confidence in the results obtained from the analysis nominated as primary [26].

4. No adjustment will be made within the principal paper(s) for other analyses including those for safety, secondary outcomes (except the CGI) [26], and health economics.

Presentation will occur as follows:

1. All analyses undertaken will be reported as far as practical (regardless of statistical significance) [65].

2. Estimated effects will be presented with unadjusted 2-sided 95% CIs and *P*-values.

3. *P*-values adjusted for multiplicity will also be presented and explained.

Interpretation will be done as indicated below:

1. Marginal interpretation of the results will be of primary interest and will be based on the size and precision of the observed differences between interventions with reference to point estimates and unadjusted 95% CIs.

2. Intervention recommendations will also take into consideration the consistency of effects

a. across any supportive intervention contrasts,

b. across sensitivity analyses, primary outcomes and time points,

c. across efficacy, safety and cost analyses, and

d. with the results of previous studies, and clinical and consumer opinions.

##### 

**Method for handling compliance** The primary analysis will be based on the intention-to-treat principle which compares the randomised intervention policies rather than the interventions *per se*[48]. Interpretation of the extent to which intervention effect estimates reflect the effects of the intervention described in the protocol requires analyses focusing on the effects of the interventions received rather than the interventions prescribed. It is recognised that per-protocol analyses have a number of limitations, most importantly, selection biases resulting from participants who are excluded not being a simple random sample of those randomised. As such, discrepancies between the conclusions of an intention-to-treat analysis and a per-protocol analysis may not reflect discrepancies between the effects of the intervention prescribed and the intervention received. Acknowledging these and other limitations, a per-protocol analysis will serve as the primary sensitivity analysis investigating the robustness of the conclusions of the primary analysis to assumptions about departures from the randomised intervention policies.

#### ***Descriptive analyses***

##### 

**Description of available data** The patterns of availability of baseline and follow-up data will be summarised overall and separately for the four interventions and for each assessment visit at the scale level. If one or more case report forms (CRFs) are available for a particular visit then the visit will be regarded as available. If one or more (non-administrative) items are available then the scale will be regarded as available. Availability of baseline and follow-up data will be summarised with differentiation of fully, or partially completed measures from those completely missing, or with sketchy detail.

The timing of baseline and follow-up data will be summarised overall and by intervention for each assessment visit in terms of the median (lower quartile, upper quartile, minimum and maximum) number of days from randomisation and the proportion falling outside guideline timeframes. Histograms of distributions will also be examined. Where assessments for a particular visit are carried out on more than one date, the timing of CFQ and SF-36PF assessments will be used to summarise visit timing. The extent to which visits are carried out on more than one date will be examined together with any further relevant details.

##### 

**Description of missing data** Where available, the reasons for missing baseline and follow-up data will be summarised overall and by intervention at the visit and scale levels. This will be done using relevant information included in the comments fields of the database. It is anticipated that such information will be available principally for visit and scale missing data.

Where the level of item-missing data is borderline between ‘minimal’ and ‘important’ (see Methods for Handling Dropouts and Missing Data), the appropriateness of prorating will be evaluated using the checks outlined by Fayers *et al*. [56]. Assumptions regarding the nature of the missing data mechanism (that is, MAR as compared to MCAR and MAR, conditional on the variables included in the substantive model as compared to additional variables) will be evaluated by looking descriptively at the statistical associations between whether or not data is missing and any potential predictors, including those generated by looking at the comments fields or the data.

##### 

**Figure 3:**
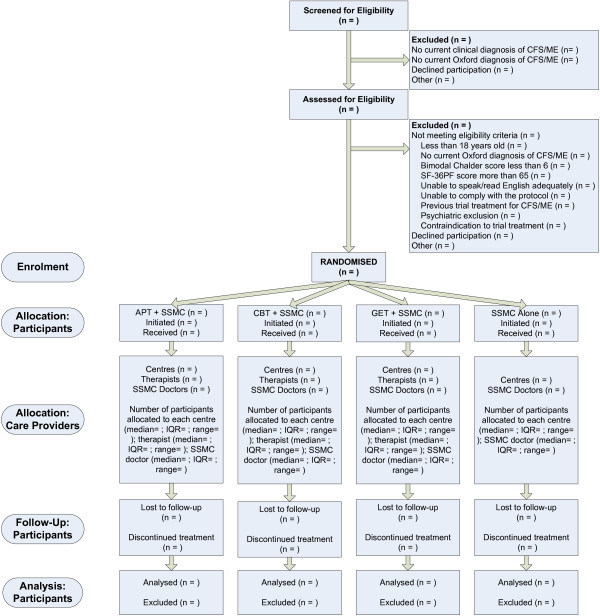
CONSORT flow diagram.

**Participant flow** Participant throughput will be summarised in a CONSORT diagram [28] including the stages of enrolment, allocation, follow-up and analysis (see Figure 3). Where available, similar summary information will also be provided on the flow of therapists and doctors from recruitment to analysis [66]. In addition to the median, lower quartile, upper quartile minimum and maximum, the arithmetic, harmonic and minimum-variance mean cluster sizes together with the standard deviation will be tabulated by intervention as these may be useful for calculating the design effect where cluster sizes are variable in size [67,68].

Any participant attending at least one session of SSMC or at least one session of APT, CBT, or GET will be regarded as having initiated their randomised intervention. The overall definition of departures from randomised intervention policy (see Departures from Randomised Intervention Policy) will be used to define an inadequate randomised intervention.

##### 

**Representativeness of sample** This will be presented within the baseline comparability tables (see Baseline Comparability of Randomised Groups).

##### 

**Baseline comparability of randomised groups** The following participant-level baseline variables will be summarised both overall and between randomised interventions:

i) Oxford criteria met (yes; no)

ii) Centre (Barts, Bristol, Edinburgh, Kings, Oxford, Royal Free)

iii) Diagnostic criteria (neither met; CDC met only; London met only; both met)

iv) Current depressive disorder (present or absent)

v) GAD (yes, no)

vi) Agoraphobia (yes, no)

vii) Panic disorder (yes, no)

viii) Fibromyalgia (met, unmet)

ix) Duration of CFS/ME since start of illness

x) Taking hypnotics, analgesics or antidepressants

xi) Number of other medications/treatments taken

xii) CFQ Score (continuous)

xiii) SF-36PF score

xiv) Age at randomisation (years) (continuous)

xv) Age at randomisation (years) (18 to 29; 30 to 39; 40 to 49; 50 to 59; 60+)

xvi) Sex (male; female)

xvii) Ethnicity (white; other, unless ‘other’ can be split further)

xviii) Marital status (married and co-habiting, single, divorced/separated/widowed)

xix) Group membership (none; self-help only; national only; both)

xx) Employment status

xxi) Health care costs

xxii) Social care costs

xxiii) Cost of lost employment

Eyeball comparisons of distributions will be carried out as a measure of the randomisation integrity.

The following therapist-level baseline variables will be summarised overall:

i) Primary professional healthcare qualification

ii) Number of calendar years between gaining primary professional healthcare qualification and start date in PACE

iii) Worked in CFS/ME or chronic pain service previously

iv) Employment grade (for health economic analysis)

Doctor variables will be summarised by:

i) Discipline (for example, psychiatrist/physician/GP)

ii) Grade (for example, Consultant/SpR/SHO)

Numbers (with percentages) for binary and categorical variables, and ordered categories plus means (and standard deviations), or medians (with lower and upper quartiles) for continuous variables will be presented. No statistical significance tests or CIs will be calculated for differences between randomised interventions on any participant-level baseline variables [69-71]. Differences in therapist-level baseline variables are expected because therapist characteristics are a component of the randomised intervention policies.

Median (lower and upper quartile) of number of participants per therapist will be reported.

##### 

**Comparison of losses to follow-up** Losses to follow-up will be reported at 13, 26, and 52 weeks by intervention and centre. Narrative summaries will be given of the reasons when known.

##### 

**Therapy and other treatment received** Summaries will be given of treatment received under the intervention policies; these will include:

i) SSMC and APT/CBT/GET received

a. Median (lower and upper quartile, minimum and maximum) number of SSMC sessions attended

a. Median (lower and upper quartile, minimum and maximum) number of APT/CBT/GET sessions attended

ii) Median (lower and upper quartile, minimum and maximum) of proportion of telephone sessions per participant

iii) Patterns of concomitant medications and treatments received - number (proportion) of participants taking hypnotics, analgesics, antidepressants (all as classified by BNF), non-pharmacological treatments, complementary and alternative medicines, up to 52 weeks.

The number and percentage of those who comply will be reported by randomised intervention within the CONSORT diagram. In addition, more detailed descriptions will be given by randomised intervention including:

i) Number (percentage) of participants attending (i) fewer than three sessions of SSMC or (ii) fewer than ten sessions of APT, CBT or GET.

ii) Number and percentage of participants initiating a trial therapy other than the one randomised.

iii) Number, percentage and details of participants receiving a trial intervention from (i) more than one therapist/doctor, (ii) a therapist/doctor from a different centre, or (iii) a therapist delivering their second therapy type.

Details of the following will be reported overall and by randomised intervention:

a) Mid-trial modifications to trial interventions and manuals.

b) Partial suspension of randomisation.

Narrative summaries will be given of the reasons for withdrawal when known.

Each primary outcome will be tabulated in a 2 × 4 table by compliance status and randomised intervention.

##### 

**Unblinding of randomised intervention** While this trial is not blinded, due to impracticability, a number of steps were taken to minimise bias arising from this. The apparent success of these steps will be assessed where possible:

1. Extent of any unblinding of the Trial Steering and Data Monitoring Committees or the blinded statisticians will be reported.

2. Extent of primary outcomes data collected over the phone will be reported by randomised intervention.

3. The degree of self-declared expectations of the trial outcome among the trial team by professional role (that is, SSMC doctor, APT/CBT/GET therapist, therapy leader, centre leader, research staff) and centre by randomised intervention was collected.

4. Participant preferences will be reported by randomised intervention.

5. Participant expectations of outcome will be reported by randomised intervention.

6. Proportion and type of discrepancies between preferred intervention and randomised intervention will be reported by randomised intervention.

#### ***Interim analyses and safety monitoring analyses***

No interim analyses were planned or have been carried out.

#### ***Analysis of fatigue and disability (co-primary outcomes)***

##### **Definition of outcome measure (including trial periods)**

The fatigue and physical disability outcomes are continuous scores defined separately at weeks 12, 24, and 52. These are the primary outcomes.

Fatigue will be measured by the Likert scores of the CFQ (possible range 0 to 33).

Physical disability will be measured by the continuous scale of the SF36-PF (possible range 0 to 100).

##### 

**Descriptive statistics for outcome measures** The distributions of the Likert Chalder fatigue scores will be presented in frequency histograms both overall and by intervention at each assessment point (baseline, weeks 12, 24, and 52). The distribution of the SF-36 physical function subscale score will also be presented in histograms both overall and by intervention at each assessment point. It is anticipated that the distributions of the Likert Chalder fatigue score and the SF-36 physical function subscale score will be approximately normally distributed. Summary statistics (minimum, maximum, mean and standard deviation, median and inter-quartile range) will be tabulated and the response profiles plotted for each continuous score both overall and by intervention at each assessment point. The response profiles over time will also be plotted by outcome and intervention.

The mean scores (Likert Chalder fatigue scores and SF-36 physical function subscale scores) within each main therapist’s caseload will be calculated by therapy (APT, CBT and GET). These means will be plotted to investigate the level of variability in participant outcomes between therapists and to examine the distribution of these summary statistics (that is, whether they are normally distributed or skewed). Differences in the mean scores within each main doctor’s caseload will also be calculated and similar plots based on these presented.

##### 

**Primary analysis (including method of analysis)** The primary analysis addressing primary objectives (1) to (5) and secondary objectives (1) and (3) will be based on the principle of intention-to-treat. If missing data are estimated using multiple imputation this analysis will be based on the intention-to-treat sample (see Trial Samples); if missing data are estimated via prorating and maximum likelihood, the analysis will be based on the available-case sample (see Trial Samples) and will exclude any participants with no follow-up data in a ‘modified ITT’ analysis. The primary outcomes of fatigue and physical disability will be analysed separately using two mixed-effects linear regressions, each including participant as a random intercept and investigating adding a random slope on time. Time (investigating the possibility of linearising across 12, 24 and 52 weeks), the time-by-intervention interaction, baseline CFQ Likert score, baseline SF-36 physical function score and the design factors (that is, centre, CDC criteria, London criteria and current depressive disorder) will be included as fixed effects. Primary interest will be in the fixed contrasts specified in Method for Handling Multiple Comparisons and Multiplicity section at 52 weeks. The statistical models used in the analysis will be reported in full.

##### 

**Clinical importance of the mean differences in primary outcomes at 52 weeks** This will be judged by reference to the trial sample SDs at baseline in this trial supported by estimates from other sources. Specifically, a difference between means of two intervention groups, at 52 weeks, of 0.3 SD will be regarded as of minimal clinical importance (a MCID) and of 0.5 SD as a clinically useful difference. From published literature on these scales these differences can be translated into 5 points on the SF-36PF, and 1.2 points on the CFQ, for minimal clinical importance and 8 points on the SF-36PF, and 2.0 points on the CFQ, for clinically useful.

##### **Baseline adjustment**

By design factors only

Not applicable

By design factors and additional factors

This is the primary analysis.

##### 

**Model assumption checks** The following assumptions will be checked:

1. Independence of residuals will be checked using the supportive analyses described in Method for Handling Other Clustering Effects section. ICC and within-cluster variance estimates will be reported.

2. Distribution of residuals and random effects will be checked visually using Q-Q plots and histograms of the residuals and by plotting the between-participant variation in participant outcomes and where appropriate the between-centre, the within-doctor but between-interventions, and the between-therapist variation in participant outcomes. Deviations from a Normal distribution would indicate a violation of model assumptions. In this event an alternative approach to the analysis would be investigated.

3. Equal variance of residuals will be checked visually using plots of the standardised residuals against the predicted values.

4. Absence of an intervention-by-centre interaction will be checked in the primary analysis by including fixed contrasts for the intervention-by-centre interaction.

Checks will be made for extreme outliers and points with high leverage. In the event that these are found, the analysis will be reported with and without these observations together with any relevant details.

##### 

**Other analyses supporting the primary analysis** A number of sensitivity analyses will be employed to examine the robustness of the conclusions of the primary analysis to:

1. Categorical responder/improver analysis.

Clinically significant improvement will be taken as a CGI participant score of 1 (very much improved) or 2 (much improved). CGI (P) scores of 3 (a little improved), 4 (no change) and 5 (a little worse) will be considered as non-improvement. CGI (P) scores of 6 (much worse) and 7 (very much worse) will be considered as deterioration. The primary analyses will be repeated replacing the primary outcomes with the CGI (P) (response versus no response versus deterioration) and using mixed-effects logistic regression.

2. Methods for handling missing data.

The primary analysis will be repeated assuming data are missing not at random (MNAR) as described in Method for Handling Dropouts and Missing Data section.

3. Choice of sample.

A per-protocol analysis will be employed using the per-protocol sample to examine the robustness of the results of the primary analysis to departures from the intended randomised intervention or eligibility criteria.

##### 

**Additional analyses** The CBT versus GET contrast will be reported, recognising its exploratory status.

Secondary objective (2) (Do different interventions have differential effects on primary outcomes?) will be addressed by extracting fixed contrasts for the outcome-type-by-intervention interaction from a bivariate mixed-effects linear regression [51,72-74] fitted with fatigue and physical disability as joint outcomes, participant as a random effect (investigating adding a random slope on time), outcome-type (physical disability versus fatigue), intervention (all contrasts specified), time (investigating linearising this effect across 12, 24 and 52 weeks), the time-by-intervention interaction, the outcome-type-by-intervention interaction, baseline CFQ Likert score, baseline SF-36 physical function score and the design factors (that is, centre, CDC criteria, London criteria and current depressive disorder) as fixed effects. These contrasts directly estimate the differences in the intervention effects between the two primary outcomes.

#### ***Analysis of secondary outcomes***

##### **Efficacy outcomes**

##### 

**Definition of outcome measures (including trial periods)** All secondary efficacy outcomes are defined separately at weeks 12, 24 and 52 unless specified otherwise (see Baseline and Outcome Measures). The PACE Scoring Protocol outlines in detail the process for calculating scores from questionnaire items and variables from case report forms. Participant-, therapist- and SSMC doctor-rated CGI are defined as ordinal variables with three categories. Participant satisfaction is defined as an ordinal variable with seven categories. The anxiety and depression subscale scores of the HADS, the Walking Test, and the total score of the Work and Social Adjustment scale are all continuous variables. However, the distribution of these is not pre-specified with the possibility that some or all may be skewed and the Walking Test may be bimodal. The number of CDC symptoms is a count variable and CDC Symptoms (1) and (8) are binary variables.

##### 

**Descriptive statistics for outcome measures** The distributions of all secondary efficacy outcomes will be presented in histograms (continuous/count) or bar charts (ordinal/binary) both overall and by intervention at each assessment point. A single table will be produced including summary statistics for all secondary efficacy outcomes by intervention and assessment point. Numbers (and percentages) or means (and standard deviations, minimums and maximums) or medians (and inter-quartile ranges, minimum and maximums) will be presented as appropriate. Summary statistics will be further plotted using line graphs for each outcome across time by intervention. The anticipated profiles have not been specified in advance. Potential variability in secondary efficacy outcomes between therapists and between doctors will be investigated using an approach similar to that outlined for the primary outcomes.

##### 

**Primary analysis (including method of analysis)** The primary analyses addressing secondary objective (3) will involve the secondary efficacy outcomes and will be based on the intention-to-treat principle. Participant will be included as a random intercept (investigating adding a random slope on time), time (investigating the possibility of linearising this effect across 12, 24 and 52 weeks) and the associated baseline variable as fixed effects and centre, CDC criteria, London criteria, and Current Depressive Disorder as fixed indicator variables. Participant-rated CGI and the participant satisfaction will be analysed using mixed-effects ordinal logistic regressions. The anxiety and depression subscale scores of the HADS, number of CDC symptoms, the Jenkins sleep scale total score, the Walking Test, and the total score of the Work and Social Adjustment scale will be analysed using mixed-effects linear regressions, unless there is evidence to suggest that these outcomes are skewed/bimodal, in which case transformation and bootstrapping will be investigated. CDC Symptoms (1) and (8) will be analysed using mixed-effects logistic regressions. The intervention and time-by-intervention contrasts fitted for the primary outcomes will be extracted for each secondary efficacy outcome as outlined in the analyses of the primary outcomes.

##### 

**Baseline adjustment** The same as that outlined for the primary outcomes

##### 

**Model assumptions checks** The following will be checked as described for the primary analysis of the primary outcomes

1. Independence of residuals

2. Distribution of residuals

3. Equal variance of residuals

4. Distribution of random effects (as appropriate)

5. Absence of an intervention-by-centre interaction

6. Extreme outliers and points with high leverage

##### 

**Other analyses supporting the primary analysis** Sensitivity analyses investigating the robustness of the conclusions of the primary analyses of the secondary efficacy outcomes will be less extensive than those described for the primary outcomes unless concern is raised by those carried out for the primary outcomes.

##### 

**Safety outcomes** These analyses will be based on the safety sample (see Trial Samples).

##### 

**Definition of outcome measures (including trial periods)** The safety of the trial interventions will be assessed using the definition of serious deterioration that was developed for monitoring safety during the course of the trial (see Outcome Measures), participant-rated adverse events defined and recorded in accordance with the protocol, and withdrawals from intervention. Serious deterioration, defined at 52 weeks, will be the primary assessment of safety. Its four components will be reported separately to enable evaluation of their relative contributions. These draw on the two adverse outcomes defined in the protocol, namely negative change on either the participant-rated CGI or the SF-36 physical function scale defined at 12, 24 and 52 weeks.

Participant-reported adverse events, including comorbid conditions which started after randomisation, are reported in terms of their relatedness to the trial intervention (events versus reactions), seriousness (non-serious versus serious) and severity (mild, moderate, severe). In addition serious adverse events are reported by the above and by their expectedness (expected versus unexpected).

Three independent assessors, initially blinded to intervention, selected by AfME and approved by the TSC, will do the following:

1. review all non-serious adverse events to determining if any should be upgraded to serious adverse events (SAEs) (masked to intervention);

2. review all SAEs to agree their classification as such (masked to intervention);

3. rate the relationship of each SAE to the randomised interventions (unmasked to intervention) (to consider whether any might be serious adverse reactions (SAR) to an intervention or suspected unexpected serious adverse reactions (SUSAR)); and

4. review all SARs and SUSARs.

Assessors will work independently of each other during the both classification periods. Where there is disagreement, consensus will be sought. Where disagreement continues, a majority vote will be taken.

##### **Descriptive statistics for outcome measures**

##### 

**Serious deterioration** Serious deterioration will be tabulated both overall and by its four components at week 52 by randomised intervention. Absolute risk difference tests will be performed between serious deterioration (yes or no) and randomised intervention.

##### 

**Adverse events** Adverse events will be tabulated separately by type (non-serious adverse events, serious adverse events, serious adverse reactions and suspected unexpected serious adverse reactions), by time (weeks 0 to 12, weeks 12 to 26, weeks 26 to 52, and overall weeks 0 to 52), and by randomised intervention. Each table will include denominators showing how many participants were in the trial at each time point by randomised intervention. The numerator will indicate the number of affected participants, and an event rate will be provided indicating the events per unit of person time so as to capture events with recurrences.

The frequency of non-serious adverse events (non-serious adverse events and non-serious adverse reactions) per participant will be tabulated by randomised intervention.

All serious adverse events will be described individually: stating randomised intervention, participant identification number, centre, sex and age, investigator's reported term, preferred term, date of onset according to the date of the randomization, duration, number of SSMC sessions, number of therapist sessions (if applicable), action taken regarding the study intervention administration, use of a corrective treatment, outcome, relationship to the study intervention in the PACE clinician’s opinion and expectedness. Where the independent scrutineers have disagreed with the PACE clinician’s opinion, the scrutineers’ views only will be reported.

Deaths will be reported as described for a serious adverse event.

All adverse events leading to withdrawal (which constitute significant adverse events) will be summarised by randomised intervention, and whether the participant withdrew from the whole trial or intervention only.

##### **Discontinuation and withdrawals from intervention**

Discontinuation and withdrawals from intervention will be listed by intervention, participant identification number, centre, who made decision for withdrawal, whether the participant withdrew from intervention or trial, the reason for withdrawal, and interval post-randomisation (in days). Reasons for discontinuation and withdrawal from intervention will be tabulated by time (week 0 to week 12, week 12 to week 26, week 26 to week 52 and week 0 to week 52), randomised intervention and reason for withdrawal.

More detailed descriptions of adverse events will be published separately.

##### 

**Primary analysis (including method of analysis)** All serious adverse events (SAEs, SARs and SUSARs combined) will be tabulated in relation to the intervention. Any doubling in harms observed between interventions will be highlighted. The percentages of participants with SAEs, SARs and SUSARS, and the three combined, as well as number of non-serious AE and percentage of participants with one or more non-serious AEs, will be reported by intervention group, including differences between groups with 95% CIs.

##### **Health economics outcomes**

**Definition of outcome measures (including trial periods) Service use and lost employment** Comprehensive data are being collected on all health, social care and other relevant services used by individual study members using a tailored version of the Client Service Receipt Inventory (CSRI). The CSRI is used at baseline and at 24- and 52-week follow-up each time covering resource use for the previous 6 months. The CSRI covers the following broad categories of information.

• Living situation/accommodation

• Education, employment and income (including benefits)

• Time off work (measured in days) and time unemployed (or retired due to illness) summing the relevant cost period (−24 to 0 weeks, 0 to 24 weeks 0 to 52 weeks)

• Use of health and social care resources

##### 

**Cost calculation** The costs of each resource item will be calculated using best available unit cost estimates [75]. The cost of APT, GET and CBT will be estimated using information on the core resource inputs involved in delivering the interventions, and estimating country-specific costs for those inputs. Costs will be calculated using data on the number of intervention sessions received by each participant.

Lost employment costs for those in employment will be calculated by combining time off work with daily earnings. For those unemployed/retired due to ill health lost employment costs will be calculated by combining this period of time with average age and gender specific earnings.

The variables derived from the CSRI will be: (i) use (yes/no) of each service, (ii) number of service contacts/days in hospital, (iii) cost of each service, (iv) in employment (yes/no), (v) days not worked, and (vi) whether benefits received (each benefit - yes/no).

##### 

**Quality adjusted life year measurement** The EQ-5D consists of five domains (mobility, self-care, usual activities, pain/discomfort, and anxiety/depression). Each of these will receive a score of 1, 2 or 3 corresponding to no problems, moderate problems and major problems. Utility scores will be attached to each health state based on these scores (a table of utility values [76] has been produced by the Centre for Health Economics, University of York). These utility scores will be used to generate QALY gains over the follow-up period.

##### 

**Descriptive statistics for outcome measures** Data will be reported on the number and percentage of participants using each service in the CSRI by intervention, at baseline and 24 and 52 week follow-up. The mean and standard deviation number of service contacts for using services will also be reported as well as the mean and standard deviation costs for all participants. The number and percentage of participants with a score of 1, 2 or 3 for each EQ-5D domain will be reported.

##### **Primary analysis (including method of analysis)**

**Cost comparisons** Regression analysis will be used to compare service costs and total costs between the four interventions which will each be represented by dummy variables. Each intervention will be used in turn as the reference category to make all relevant comparisons.

##### 

**Predictors of cost** Participant characteristics will be used in a regression model to explain differences in baseline costs. We will test the hypothesised associations with both healthcare and societal costs, as well as using multivariable modelling of other possible predictors identified from univariate analyses. Subsequent regression models will be used to explain variations in follow-up costs, and these will also include clinical characteristics from preceding periods. Two types of regression model will be used. First, we will construct ordinary least squares models, with bootstrapping used to produce reliable 95% CIs around the regression coefficients. Second, we will construct generalised linear models with a log link and gamma distribution to account for the skewness that is likely in the costs data.

Independent variables will include demographic characteristics (such as age, gender and marital status), year of randomisation, clinical variables (such as fatigue score, disability, depression, anxiety) and benefits status (whether receiving benefits and whether benefits are in dispute).

##### 

**Cost-effectiveness analysis** Cost-effectiveness will be assessed by linking data on service cost differences and outcome (fatigue and physical disability) differences [77]. If any intervention has significantly lower costs and significantly better outcomes then it will be deemed to be more cost-effective. If costs are significantly higher and outcomes significantly better or if there is uncertainty in these findings (indicated by the CIs) then we will use the net benefit approach and cost-effectiveness acceptability curves to assess cost-effectiveness. Cost-effectiveness results will be plotted on a cost-effectiveness plane. This will involve producing estimates of cost and outcome differences from 1,000 bootstrapped re-samples of the original data. Such planes will be produced for each combination of two-way group comparisons. The plane will inform us as to the probability that an intervention has either (i) lower costs and better outcomes, (ii) lower costs and worse outcomes, (iii) higher costs and better outcomes or (iv) higher costs and worse outcomes than each comparator.

##### 

**Cost-utility analysis** This will be conducted in the same way as the cost-effectiveness analysis but will use quality adjusted life years (derived from the EQ-5D) as the outcome measure.

##### 

**Predictors of cost-effectiveness/cost-utility** The net-benefit approach allows multivariable analyses of economic data. This will enable us to identify predictors of cost-effectiveness and cost-utility. This will be done using regression models as described above. In particular we hypothesise that age and gender will predict cost-effectiveness and cost-utility.

##### 

**Baseline adjustment** The predictors of the cost regression model will be adjusted by the CSRI baseline outcome data.

##### 

**Model assumptions checks** Cost data are usually skewed and if this results in similarly skewed residuals then the standard linear model is inappropriate. The distribution of the regression residuals will be checked visually and if the distribution is non-normal we will use bootstrapping with 10,000 resamples to estimate 95% CIs around the cost differences (CIs will be based on the percentile or bias-corrected method depending on the level of bias observed in the model.) The assumption of independent residuals will be checked by bootstrapping at the therapist level.

##### 

**Other analyses supporting the primary analysis (including sensitivity analyses)** Sensitivity analyses will be carried out on two aspects of the analyses to assess the robustness of the findings. The effect of each of these alternative approaches on mean total societal costs at 12 months and subsequent cost-effectiveness calculations based on these costs will be explored in turn.

The main analyses will use an informal care unit cost based on the replacement method (where the cost of a homecare worker is used as a proxy for informal care). We will alternatively use a zero cost and a cost based on the national minimum wage for informal care. We will also conduct sensitivity analyses around the costs attached to lost employment.

The estimated costs of APT, GET and CBT will be increased and decreased by 50% to see how sensitive the costs, cost-effectiveness and cost-utility findings are to these variables.

#### ***Subgroup analyses***

Exploratory sub-group analyses are planned to investigate whether intervention effects differ between those meeting and not meeting the CDC criteria or London criteria and between those with or without a depressive disorder at the point of randomisation.

#### ***Software***

The data has been entered and checked during the course of the trial in a customised Microsoft Access [78] database. Once the database is locked, the data will be transferred into Stata [79]. It is anticipated that the analyses will be carried out primarily within Stata [79], although MLwiN [80] and other statistical packages may be used as necessary. The most up-to-date version available will be used in each case.

## Abbreviations

AE: adverse event; AfME: action for myalgic encephalomyelitis or encephalopathy; APT: adaptive pacing therapy; CBT: cognitive behaviour therapy; CDC: Centers for Disease Control, Atlanta, Georgia, USA; CGI: clinical global impression; CFQ: Chalder fatigue questionnaire; CFS: chronic fatigue syndrome; CI: confidence interval; CONSORT: Consolidated Standards of Reporting Trials; CPMP: Committee for Proprietary Medicinal Products; CRF: case report form; CSRI: client services receipt inventory; DMC: data monitoring committee; DMEC: data monitoring and ethics committee; EAS: exercise and activity scale; EuroQoL: European quality of life scale (EQ-5D); GAD: generalised anxiety disorder; GET: graded exercise therapy; GP: general practitioner; HADS: hospital anxiety and depression scale; ICC: intraclass correlation coefficient; ICH: International Conference on Harmonisation; ID: identification; IQR: interquartile range; ISRCTN: international standard randomised controlled trial number; ITT: intention to treat; MAR: missing at random; MCAR: missing completely at random; ME: myalgic encephalomyelitis or encephalopathy; MH&N: Mental Health & Neuroscience; MNAR: missing not at random; NHS: National Health Service, UK; NICE: National Institute for Health and Clinical Excellence; PACE: pacing, graded activity, and cognitive behaviour therapy: a randomised evaluation; PHQ-15: physical health questionnaire - 15 items; QALY: quality adjusted life year; Q-Q: quantile-quantile; RCT: randomised controlled trial; SAE: serious adverse event; SAP: statistical analysis plan; SAR: serious adverse reaction; SD: standard deviation; SF-36: short form - 36 items; SHO: senior house officer; SIQ: symptom interpretation questionnaire; SpR: specialist registrar; SSMC: standardised specialist medical care; SUSAR: suspected unexpected serious adverse reaction; TSC: trial steering committee; WSAS: work and social adjustment scale.

## Competing interests

PDW has done voluntary and paid consultancy work for the United Kingdom Departments of Health and Work and Pensions and Swiss Re (a reinsurance company), and led a randomised controlled trial about graded exercise therapy funded by the Linbury Trust. TC has received royalties from Sheldon Press and Constable and Robinson. MS has done voluntary and paid consultancy work for the United Kingdom government, has done consultancy work for the insurance company Aegon and has received royalties from Oxford University Press. RW, LP, PM, ALJ, JCD, HB, and KAG declare that they have no conflicts of interests.

## Authors’ contributions

The principal investigators (PDW, TC, and MS) designed the study and obtained funding. The analysis strategy group, chaired by MS, consisted of all the authors of this report including HB, TC, JCD, KG, ALJ, PM, LP, RW and PDW. The statistical analysis plan was jointly written by the analysis strategy group and approved by the trial management group, trial steering committee and data monitoring and ethics committee before the analysis was started. ALJ, LP, and RW were the trial statisticians who participated in the design of the study, while KG and ALJ were the trial statisticians who participated in the main statistical analysis. RW was the lead writer of the analysis plan and the paper, LP and PM were co-writers and ALJ was lead methodologist. The trial managers were JCD initially, then HB. All authors read and approved the final manuscript.
